# Epidural labor analgesia in a pregnant woman with symptomatic recurrence of myelin oligodendrocyte glycoprotein antibody-associated disease: a case report

**DOI:** 10.1186/s40981-025-00829-1

**Published:** 2025-11-05

**Authors:** Satoshi Toyama, Sooyeon Han

**Affiliations:** Department of Anesthesiology, Graduate School of Medical and Dental Sciences, Institute of Science Tokyo, 1-5-45Bunkyo-Ku, Yushima, 1138510 Japan

**Keywords:** Myelin oligodendrocyte glycoprotein antibody-associated disease, Demyelinating disorder, Labor analgesia, Neuraxial anesthesia, Obstetric anesthesia

## Abstract

**Background:**

Myelin oligodendrocyte glycoprotein antibody-associated disease (MOGAD) is a demyelinating disorder that may relapse during pregnancy. The safety of neuraxial anesthesia in such patients remains unclear.

**Case presentation:**

We describe a 32-year-old multiparous woman with MOGAD who developed intractable hiccups due to new medullary lesions at 35 weeks of gestation. Despite steroid pulse therapy, symptoms persisted. At 36 weeks and 2 days, premature rupture of membranes occurred, and hiccups worsened with labor pain. After informed consent, labor epidural analgesia was initiated using low-concentration levobupivacaine with minimal fentanyl. Pain relief coincided with the reduction of hiccups. Vaginal delivery of a male infant was uneventful. Her hiccups resolved by postpartum day 4, and magnetic resonance imaging showed improvement in the lesion. She was discharged on postpartum day 7 without neurological worsening.

**Conclusions:**

Labor epidural analgesia provided effective pain control and was associated with symptom relief in a parturient with active MOGAD.

## Background

Myelin oligodendrocyte glycoprotein antibody-associated disease (MOGAD) is a demyelinating disorder of the central nervous system that has been increasingly recognized as distinct from multiple sclerosis (MS) and aquaporin-4 antibody-positive neuromyelitis optica spectrum disorder (NMOSD) [1]. MOGAD is characterised by recurrent optic neuritis, myelitis, and brainstem involvement, with variable neurological sequelae [1–3]. Pregnancy modulates immune responses, and relapse may occur during gestation or the postpartum period [4–6].

While neuraxial anesthesia is commonly used in obstetric practice, its use in patients with demyelinating diseases remains controversial. In MS and NNOSD, the safety of neuraxial anesthesia in asymptomatic patients has been demonstrated in obstetric settings [5, 7, 8]. However, evidence in MOGAD remains limited, and concerns persist when new spinal cord or brainstem lesions are present [9, 10]. Stress during labor may exacerbate neurological symptoms [11], but general anesthesia for cesarean delivery carries its own risks [12]. Therefore, the choice of anesthetic technique requires careful consideration and a shared decision-making process.

We report a case of active MOGAD with medullary and spinal cord involvement complicated by fetal growth restriction (FGR), where labor epidural analgesia provided effective pain relief and coincided with improvement of intractable hiccups.

Written informed consent for publication of this case report and all accompanying images was obtained from the patient.

## Case presentation

A 32-year-old woman (152 cm, 52 kg), gravida 2 and para 1, with MOGAD, gestational diabetes (GDM), and FGR, was referred to our hospital at 35 weeks of gestation. MOGAD first manifested 6 years earlier with diplopia and headache; MOG-IgG positivity confirmed the diagnosis. She experienced two relapses before her first pregnancy, which was uneventful and ended in vaginal delivery without epidural analgesia.

**Fig. 1:**
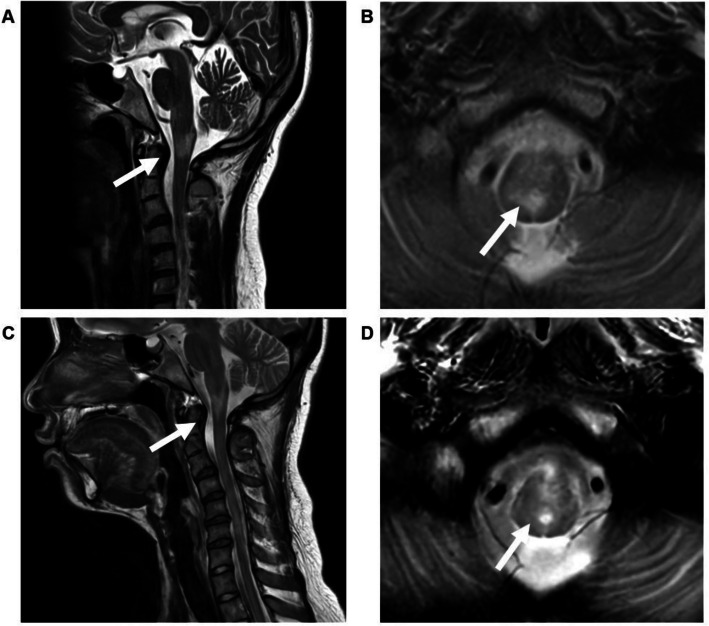
Magnetic resonance imaging (MRI) findings. Sagittal (**A**) and axial (**B**) T2-weighted images at 35 weeks of gestation show hyperintense lesions from the lower medulla to the upper thoracic spinal cord. The prepartum hyperintense lesions show improvement, and no new abnormalities are identified in sagittal (**C**) and axial (**D**) T2-weighted magnetic resonance images obtained after delivery. Arrows indicate the affected areas

In the current pregnancy, relapse occurred two months after conception with left optic neuritis and brainstem lesions, treated with methylprednisolone pulse therapy and prednisolone 10 mg/day. At five months’ gestation, she had a further relapse with right optic neuritis and periventricular lesions around the third ventricle. After repeated methylprednisone pulse therapy, her maintenance prednisolone was increased to 12.5 mg/day. At 35 weeks of gestation, she developed persistent hiccups. Magnetic resonance imaging (MRI) revealed new T2-weighted hyperintense lesions extending from the lower medulla to the upper thoracic cord (Fig. 1). Because of the risk of respiratory depression and quadriparesis, she was transferred to our hospital.

On admission, she presented with persistent and continuous hiccups, right temporal tenderness, and hyperreflexia in all extremities, without motor or sensory deficits. The hiccups were so frequent that she was unable to take any oral intake, resulting in upper gastrointestinal bleeding, and she required intravenous fluid and nutritional support. Thus, she received intravenous methylprednisolone 1,000 mg/day for three days. However, since hiccups persisted, the treatment was extended to 5 days, followed by oral prednisolone 25 mg/day as maintenance therapy. GDM, previously diet-controlled, required insulin therapy after the initiation of methylprednisolone pulse therapy, and blood glucose levels remained well-controlled thereafter. Hiccup frequency gradually decreased with steroid therapy, improving from constant symptoms to several episodes per hour. Upper gastrointestinal bleeding resolved, and she was able to tolerate a liquid diet.

Her first pregnancy ended in vaginal delivery without epidural analgesia. However, in the current pregnancy, based on her prior painful delivery experience, she strongly requested labor epidural analgesia. At 36 weeks and 2 days of gestation, premature rupture of membranes occurred. As uterine contractions and pain began, her hiccups simultaneously worsened again to once every few minutes. Although cesarean delivery was considered as an alternative, severe hiccups could not be adequately controlled by neuraxial anesthesia alone, implying general anesthesia would be required. General anesthesia was considered less favorable due to maternal risks and neonatal depression. Moreover, if neuraxial anesthesia were to be used for cesarean delivery, epidural analgesia for vaginal delivery would represent a similarly valid, potentially safer choice. After these considerations were thoroughly discussed with the patient, she consented to epidural analgesia.

Peripartum steroid cover was initiated with an intravenous bolus of hydrocortisone 100 mg followed by continuous infusion of hydrocortisone 200 mg over 24 h [13]. An epidural catheter was inserted from the L3–L4 intervertebral space at 4 cm cervical dilatation with 70% effacement, moderate consistency, mid-position, and fetal station −2, corresponding to a Bishop score of 7. Her pain score was nine on the numerical rating scale (NRS). After an initial bolus of 10 mL of 0.1% levobupivacaine and 50 μg fentanyl, epidural analgesia was continued using programmed intermittent epidural bolus (PIEB) with 0.06% levobupivacaine with fentanyl 2 μg/mL, set at 8 mL per bolus with a lockout interval of 50 min. Following initiation of epidural analgesia, hiccups decreased in parallel with pain relief, returning to several episodes per hour. At full cervical dilatation, her breakthrough pain was managed with 50 μg fentanyl and 10 mL of 0.1% levobupivacaine, achieving NRS 0. Vaginal delivery was uneventful. The male neonate weighed 1,847 g, with Apgar scores of 8 and 7 at 1 and 5 min, and umbilical arterial pH 7.298. The epidural catheter was removed 2 h after delivery once postpartum hemostasis and hemodynamic stability were confirmed.

After delivery, hiccups decreased to several times per day and disappeared by postpartum day 4. Oral intake normalised. Some tendon reflexes improved, though hyperreflexia persisted, and a new right Babinski reflex appeared. Postpartum MRI demonstrated improvement of the medullary lesion (Fig. 1). She continued oral prednisolone 25 mg/day postpartum [14] and was discharged on postpartum day 6 without relapse.

## Discussion

MOGAD is a rare autoimmune demyelinating disorder distinct from MS and NMOSD [1]. Unlike MS, where pregnancy is generally protective [15], MOGAD may relapse during pregnancy and postpartum [6]. Our patient experienced multiple relapses during pregnancy, the last involving the medulla and spinal cord, presenting as intractable hiccups. This symptom is consistent with the involvement of medullary structures such as the nucleus tractus solitarius and adjacent respiratory centers.

The safety of neuraxial anesthesia in patients with demyelinating diseases is not well established. In pregnant women with MS, there has long been debate regarding the safety of neuraxial anesthesia. However, accumulating evidence suggests that epidural anesthesia and analgesia during labor do not increase relapse rates or worsen long-term neurological outcomes, provided patients are in remission at the time of delivery [7, 8]. Conversely, intrathecal local anesthetic administration is sometimes regarded with more caution, as demyelinated axons may be more vulnerable to neurotoxic effects [9]. While epidural anesthesia is considered to be relatively safe in pregnant women with NMOSD [5], several case reports have described neurological worsening or relapse after spinal anesthesia in patients with NMOSD [16–18]. In pregnant women with MOGAD, much less evidence is available. Relapses may occur both during pregnancy and postpartum [6], in contrast to MS, where pregnancy itself is considered protective [15]. Moreover, MOGAD lesions are often more severe, longitudinally extensive, and acutely symptomatic, as seen in our case with a medullary lesion causing intractable hiccups [19]. These features imply that data from MS cannot be directly applied to MOGAD.

General anesthesia would have avoided neuraxial exposure but carried specific risks in pregnancy, including concerns regarding neuromuscular blockade in demyelinating disorders–such as the risk of hyperkalemia with depolarizing neuromuscular blockers and the potential for prolonged action with non-depolarizing agents– [9, 20, 21], the potential for neonatal depression due to transplacental transfer of anesthetic agents in the context of prematurity and FGR, and the systemic stress of intubation and extubation. On the other hand, neuraxial analgesia, if carefully titrated, offered the benefit of reducing maternal stress and pain, which might otherwise exacerbate neurolocal symptoms [11].

In this case, the patient had symptomatic recurrence with a new spinal cord lesion, making the decision more complex. Labor-related stress was considered a potential aggravating factor, whereas cesarean delivery under general anesthesia would have posed maternal and neonatal risks. In the present case, the patient strongly desired labor epidural analgesia, partly because of her painful first delivery without neuraxial techniques. Although neuraxial anesthesia carries theoretical risks in demyelinating diseases, avoiding labor analgesia could have resulted in exacerbation of her symptoms due to stress and pain. Indeed, her hiccups worsened as labor progressed and improved after analgesia was established, suggesting that adequate pain control may have contributed to symptom relief. This aligns with literature from MS, in which epidural analgesia has been shown to be safe [5, 8, 9]; however, it extends these findings into the less well-studied population of MOGAD.

In this case, peripartum steroid coverage was administered according to established anesthesia guidelines [11], and maintenance prednisolone therapy was continued after delivery in line with recent recommendations for MOGAD management [14].

Thus, MS data provide a framework, but MOGAD requires individualized anesthetic planning that balances maternal, fetal, and anesthetic risks. Prospective studies are unlikely given the rarity of MOGAD, but accumulating case-based evidence may contribute to the development of safer anesthetic strategies for these patients.

In conclusion, epidural labor analgesia may be a viable option in pregnant patients with active MOGAD, provided that careful assessment, through counselling, and vigilant management are ensured.

## Data Availability

The accompanying images in this case report are available from the corresponding author upon reasonable request.

## Abbreviations

MOGAD: Myelin oligodendrocyte glycoprotein antibody-associated disease
MS: Multiple sclerosis
NMOSD: Neuromyelitis optica spectrum disorder
FGR: Fetal growth restriction
GDM: Gestational diabetes
MRI: Magnetic resonance imaging
NRS: Numerical rating scale
PIEB: Programmed intermittent epidural bolus
